# Comorbidity in Lichen Planus: A Retrospective Population-Based Case–Control Study in Sweden

**DOI:** 10.3390/life16040541

**Published:** 2026-03-25

**Authors:** Hilda Odell, Sandra Jerkovic Gulin, Oliver Seifert

**Affiliations:** 1 The Faculty of Medicine and Health Sciences, Linkoping University, 58183 Linköping, Sweden; 2 Department of Dermatology and Venereology, Ryhov County Hospital, Sjukhusgatan, 55305 Jönköping, Sweden; oliver.seifert@liu.se; 3 Division of Cell Biology, Department of Biomedical and Clinical Sciences, The Faculty of Medicine and Health Sciences, Linkoping University, 58183 Linköping, Sweden

**Keywords:** lichen planus, epidemiology, case–control studies, comorbidity, thyroid disease, metabolic syndrome, malignancy, Sweden, register study, positive predictive value

## Abstract

Lichen planus (LP) is a chronic immune-mediated inflammatory disease of unknown etiology affecting the skin and mucous membranes and is frequently associated with comorbid conditions, although data from Swedish populations remain limited. This retrospective population-based case–control study included all registered citizens in Region Jönköping, Sweden, between 2013 and 2022, to examine comorbidities, estimate prevalence and incidence, assess diagnostic validity of ICD-10 coding (L43), and evaluate treatment patterns. Incidence and prevalence were calculated, demographic and treatment characteristics were described, and diagnostic validity was assessed through independent medical record review of 70 randomly selected cases to determine positive predictive value (PPV). Associations between LP and predefined comorbidities were analyzed using binomial logistic regression adjusted for age and sex. Among 361,812 individuals, prevalence was 235.5 and incidence 19.6 per 100,000 inhabitants. The PPV of the LP diagnosis was 78.6%, yielding an adjusted prevalence of 184.9 per 100,000 inhabitants. Over one third of prevalent patients received topical therapy, primarily corticosteroids. LP was significantly associated with thyroid, malignant, metabolic, and autoimmune conditions. LP is relatively uncommon, ICD-10 coding shows acceptable validity, and its association with clinically relevant comorbidities highlights the need for comprehensive patient assessment.

## 1. Introduction

Lichen planus (LP) is an inflammatory mucocutaneous disease with heterogeneous clinical presentation. The most commonly affected sites are the skin (cutaneous LP, CLP) and oral mucosa (oral LP, OLP), although appendages and other mucous membranes such as the conjunctiva, esophagus, and genitalia may also be involved [1].

LP pathogenesis involves a T-cell–mediated autoimmune response, particularly cytotoxic CD8+ T cells targeting basal keratinocytes [2]. In CLP, the inflammatory milieu includes cytokines such as interleukin-21 (IL-21) and interferon-γ (IFN-γ), which appear to play key roles [3].

The etiology remains uncertain but likely reflects an interaction between genetic susceptibility and environmental triggers. Familial aggregation and HLA-related polymorphisms support a hereditary component [1,2]. Reported triggers include medications, psychosocial stress, depression, anxiety, and a possible—though inconclusive—association with hepatitis C virus (HCV) infection [2].

Global LP prevalence ranges from 0.22% to 5% [4,5,6]. The wide variation in the reported prevalence of LP may largely be explained by methodological differences between studies, but it may also reflect geographic variation and differences in the inclusion of LP subtypes. For example, an older Swedish study based on clinical sampling rather than registry data, which specifically examined oral lichen planus (OLP), reported a prevalence of approximately 1.3% [7]. Women constitute the majority of affected individuals, accounting for 60–75% of OLP [7,8] and about 50% of CLP cases [9]. Mean age at diagnosis is 50–60 years for OLP [7,8] and 40–45 years for CLP [9].

LP is generally chronic, although CLP often remits spontaneously after 1–2 years, with frequent recurrences [9]. Treatment mainly consists of topical corticosteroids [1,10]; refractory cases may require systemic corticosteroids or phototherapy [1].

LP imposes substantial psychological burden and negatively affects quality of life due to pain, pruritus, ulceration, and hygiene impairment, often leading to psychological distress [11].

Patients with LP show increased risk of comorbidities. A German population-based study reported higher prevalence of metabolic syndrome, liver and thyroid disease, malignancies, depression, and other autoimmune or inflammatory disorders including psoriasis, vitiligo, and inflammatory bowel disease [10].

Investigating LP comorbidities may improve understanding of pathophysiology and treatment strategies, supporting better clinical management and quality of life. Epidemiological studies across populations are therefore needed.

This study aims primarily to assess associations between LP and comorbid conditions in Region Jönköping, Sweden. Secondary objectives include estimating LP prevalence, evaluating validity of ICD-10 coding, and mapping treatment patterns to identify current clinical strategies and potential gaps in management. It should be noted that oral lichen planus was not included in this study, as the analysis was restricted to ICD-10 code L43 and did not capture cases coded under oral mucosal disease codes (e.g., K13.1).

## 2. Methods

This retrospective case–control study was based on routinely collected health data from *Cosmic R8*, the electronic medical record system implemented across Region Jönköping. The dataset covered the entire regional population between 1 January 2013 and 31 December 2022, thereby including both healthcare users and individuals without recorded encounters during the period.

The study design was retrospective and case–control in nature. Cases were defined as individuals with a recorded ICD-10 diagnosis of LP, including L43.0 hypertrophic LP, L43.1 bullous LP, L43.3 subacute/erosive LP, L43.8 other LP, and L43.9 LP unspecified. Diagnoses coded under K13.1 (coded oral LP) were not included in the case definition. The control group comprised all other residents of Region Jönköping without any of the specified LP codes. Age at inclusion was defined differently between the two cohorts: for cases, the age at first LP diagnosis was used, whereas for controls, age was recorded at the end of the study period. Gender was collected for all participants.

To investigate comorbidity patterns, 95 ICD-10 diagnoses were preselected based on evidence from previous literature on LP and associated conditions. These conditions were systematically assessed in the medical records of both cases and controls. Prior to analysis, the dataset was pseudo-anonymised, and only sex and age were retained as identifiable variables.

Information on healthcare utilization was also retrieved. For each encounter where LP diagnoses were recorded, the specialty of the treating physician as well as any prescribed medications were documented.

To evaluate diagnostic validity, a validation study was conducted by two dermatologists. The sample size for the validation study was determined based on the desired precision of the PPV estimate. Assuming a PPV of approximately 80%, review of 70 records allows estimation of the PPV with a 95% confidence interval of approximately ±10 percentage points. 70 records were randomly selected from the full case set of LP-coded patients (n = 851). Each reviewer applied predefined clinical and histopathological criteria to determine whether the ICD-10 coding corresponded to a true diagnosis of LP. Histopathological features included interface dermatitis with saw-tooth acanthosis, orthokeratosis with hypergranulosis, a band-like lymphocytic infiltrate, pigment incontinence, basal keratinocyte degeneration, and Civatte (cytoid) bodies. Clinical features assessed included pruritus, violaceous polygonal papules, the presence of Wickham striae, typical anatomical distribution (e.g., wrists, distal lower extremities, feet, or presacral region), Koebner phenomenon, nail involvement such as onychodystrophy, mucosal lesions, and family history of lichen planus [12]. In situations where the reviewers initially disagreed, consensus was reached through discussion. Inter-rater agreement prior to consensus was not formally quantified with a kappa statistic, as the primary aim of the validation was to determine the final PPV of the ICD-10 coding. Cases in which the ICD-10 code was supported by the medical record were classified as confirmed (true positives), while those that failed to meet the diagnostic criteria were categorized as miscoded (false positives).

Ethical considerations

This retrospective, register-based study was conducted using pseudonymised data. Consequently, written informed consent was not deemed necessary under the prevailing ethical framework. Data processing was performed in compliance with applicable data protection legislation (GDPR). The project has been approved by the Research Data Centre (FDC) in region Jönköping as well as by the Swedish Ethical Review Authority (Dnr 2023-03220-01).

Statistical analysis

The positive predictive value (PPV) of the ICD-10 coding for LP was calculated, together with a 95% confidence interval (CI). This PPV was subsequently used to adjust the prevalence and incidence estimates of LP in the study population. Age- and sex-stratified incidence and prevalence rates were computed by relating the number of identified cases within each demographic category to the corresponding population denominator, expressed per 100,000 individuals. Statistical significance between population proportions was evaluated using a two-sample Z-test. For comparisons involving small cell counts (expected counts < 5), Fisher’s exact test was applied instead. To explore associations between LP and comorbid conditions, binominal logistic regression models were employed to estimate odds ratios (OR) with 95% CI, adjusting for age and sex (adjusted odds ratio, aOR). All statistical analyses were performed in SPSS (version 31, IBM Corp., Armonk, NY, USA).

## 3. Results

Epidemiology

**Figure 1:**
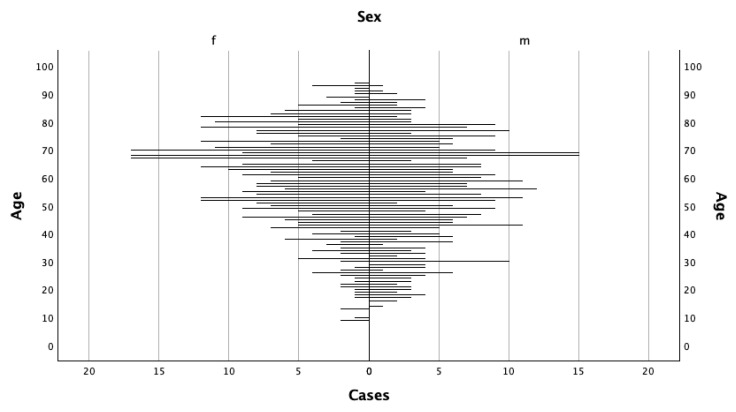
Population pyramid of prevalent cases by age and sex in 2022 (f = female, m = male).

A total of 361,812 people were included in this study, comprising 851 persons identified as prevalent LP cases (0.2%) and 360,961 controls (99.8%). Within this cohort, a total of 708 patients met criteria for incident LP during the study period between 2013 and 2022, whereas the remaining patients represented previously diagnosed individuals. An overview of the study cohort, with incident cases, is provided in Table 1. The median age among LP cases was 58 years, with the largest proportion of patients (21.2%) in the 60–69-year age range. In contrast, controls had a median age of 41 years, and the greatest share (21.6%) were individuals younger than 18 years. Sex distribution did not substantially differ between the groups, with comparable proportions of males and females observed among cases and controls. Figure 1 illustrates the age and sex specific distribution of LP cases using a population pyramid. Both men and women demonstrated a peak in incidence at approximately 70 years of age.

**Figure 2:**
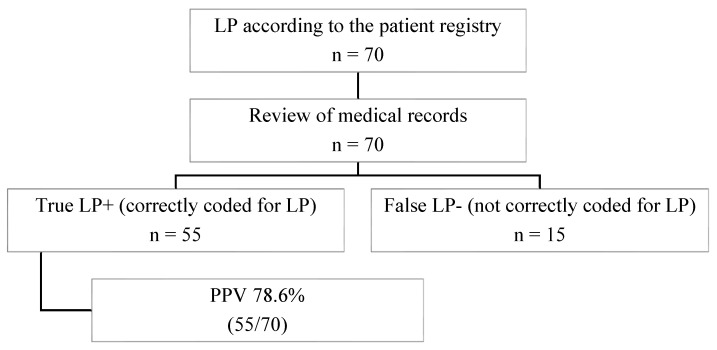
Flowchart illustrating the selection and validation process of patients with lichen planus (LP) included in the study cohort. The validation of LP diagnosis, based on ICD-10 code L43 during 2013–2022, yielded a positive predictive value (PPV) of 78.6%.

Among the 70 reviewed medical records, 55 diagnoses were confirmed as true LP, corresponding to a PPV of 78.6% (Figure 2). In 2022, the crude prevalence of LP was 235.3 per 100,000 inhabitants, based on the 851 identified cases. When adjusted according to the PPV, the prevalence was estimated at 184.9 cases per 100,000 inhabitants. The adjusted prevalence was slightly higher among females (193.8 per 100,000) than males (176.5 per 100,000). During the study period, the crude annual incidence of LP in Region Jönköping averaged 19.6 cases per 100,000 inhabitants. The PPV-adjusted annual incidence rate was 15.4 per 100,000 inhabitants, with female and male rates of 16.0 and 14.8 per 100,000, respectively.

**Figure 3:**
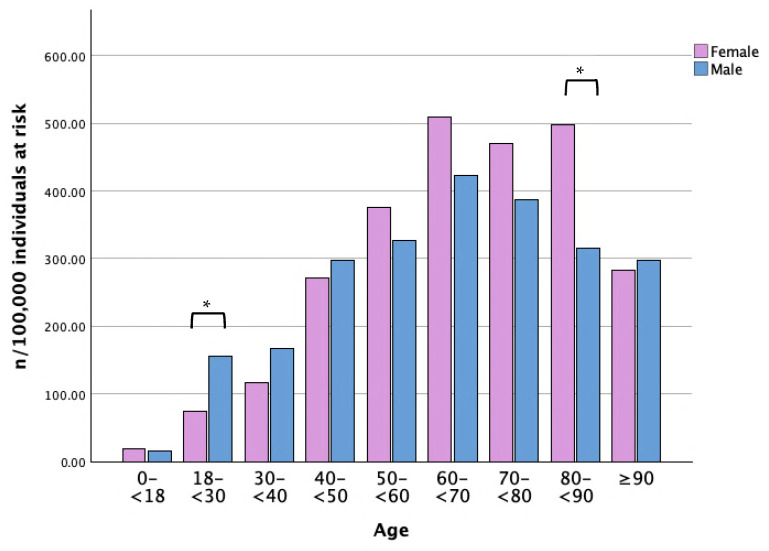
Prevalence of LP per 100,000 individuals at risk in 2022, stratified by age and sex—normalized to the population of Region Jönköping (n = number of cases, LP = lichen planus). * = *p*-value < 0.05.

In 2022, LP prevalence peaked among women aged 60–69 years (508.8 per 100,000) as well as among men (423.6 per 100,000) (Figure 3). Significantly higher prevalence was observed among women aged 80–90 years, whereas men demonstrated a significantly higher prevalence in younger age groups (18–30 years) (Figure 3).

**Figure 4:**
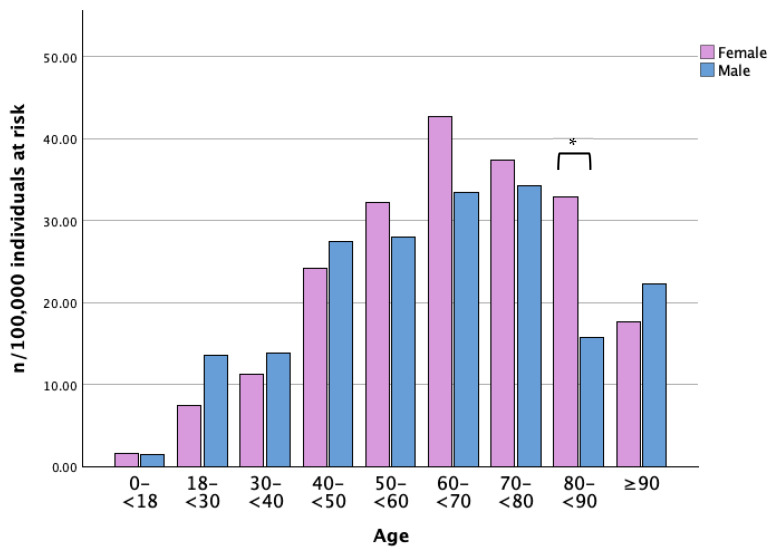
Annual mean incidence of LP per 100,000 individuals at risk between 2013 and 2022, stratified by age and sex—normalized to the population of Region Jönköping (n = number of cases, LP = lichen planus). * = *p*-value < 0.05.

Between 2013 and 2022, the highest incidence rates of LP were observed among women aged 60–69 years (42.7 per 100,000 per year) and men aged 70–79 years (34.4 per 100,000 per year), as shown in Figure 4.

**Clinical subtypes and treating physicians**

The majority of confirmed LP cases among prevalent cases were classified as unspecified LP (77.2%), while ulcerative, erythematous, or atrophic variants represented the second most frequent clinical subtype (23.6%) (Table 2). Dermatologists accounted for the majority of initial LP diagnosis (72.5%), whereas primary care physicians represented the second most common diagnostic specialty (16.1%) (Table 3).

**Therapy**

An overview of treatment patterns among patients with prevalent lichen planus in 2022 is presented in Table 4. Topical therapies were the most frequently prescribed treatment, administered to 36.9% of cases. Topical corticosteroids constituted the main therapeutic approach (30.6%). Systemic treatments were used in 4.3% of cases, most commonly systemic corticosteroids (3.5%). Notably, 57.1% of the cohort did not receive any documented LP-specific treatment. No treatment was not associated with specific subtypes, sex, age groups, or treating specialties.

**Comorbidity**

After adjusting for age and sex, patient with LP showed significantly higher odds of several comorbid conditions compared with controls (Table 5). The strongest associations among malignancies were observed for cancers of the vulva/vagina (aOR 9.4; 95% CI 2.9–29.9), penis (aOR = 10.4; 95% CI 3.2–33.2), and lip/oral cavity (aOR 7.1; 95% CI 3.8–13.4). Metabolic conditions such as hypertension (aOR = 2.0; 95% CI 1.7–2.4), dyslipidemia (aOR 1.9; 95% CI 1.6–2.2), obesity (aOR 1.6; 95% CI 1.3–1.9), and diabetes mellitus type 1 (aOR 1.9; 95% CI 1.3–2.8) and type 2 (aOR 1.8; 95% CI 1.4–2.1) were also more common in the LP group.

Psychiatric disorders including depression (aOR 1.4; 95% CI 1.2–1.6), persistent mood disorder (aOR 2.4; 95% CI 1.3–4.5), and OCD (aOR 1.9; 95% CI 1.0–3.4) were significantly associated with lichen planus. Elevated odds were also found for autoimmune diseases such as Sjögren’s syndrome (aOR 3.0; 95% CI 1.7–5.2), dermatomyositis (aOR 12; 95% CI 3.8–38.2), and systemic lupus erythematosus (aOR 2.8; 95% CI 1.1–7.6).

Lifestyle and cardiovascular conditions like smoking (aOR 1.7; 95% CI 1.3–2.1), angina pectoris (aOR 1.6; 95% CI 1.2–2.1), ischemic heart disease (aOR 2.0; 95% CI 1.6–2.5), and arteriosclerosis (aOR 2.3; 95% CI 1.4–3.6) were significantly more frequent. Infections including oral candidiasis (aOR 2.8; 95% CI 2.3–3.3) and genital candidiasis (aOR 2.5; 95% CI 1.9–3.2), as well as dermatologic conditions such as psoriasis (aOR 3.3; 95% CI 2.6–4.1), leukoplakia of the vulva/vagina (aOR 12.7; 95% CI 8.7–18.4), and lichen sclerosis (aOR 11.9; 95% CI 9.4–14.9), showed markedly increased odds. Finally, thyroid disorders were also overrepresented, particularly hypothyroidism (aOR 1.6; 95% CI 1.3–2.1). No significant associations were seen for hepatitis B or C.

Table 6 lists the conditions for which no prevalent cases of LP were identified. Consequently, no comorbidity analysis could be performed for these diagnoses.

## 4. Discussion

Discussion

This population-based study evaluated comorbidities among 851 individuals with LP compared to 360,961 controls in Region Jönköping. LP was associated with multiple systemic conditions, including selected malignancies, metabolic disorders, autoimmune diseases, cardiovascular disease, and depression. The study also estimated population prevalence, assessed diagnostic validity of ICD-10 coding, and characterized treatment patterns. The crude prevalence was 0.24%, decreasing to 0.18% after adjustment for a PPV of 78.6%. Topical corticosteroids were the most frequently prescribed treatment. Collectively, these findings expand knowledge of the epidemiology, diagnostic accuracy, and clinical management of LP.

Prevalence and Incidence

The prevalence of LP in 2022 was 235.3 per 100,000 (0.24%), and 185 per 100,000 (0.18%) after adjustment. This falls just below the globally reported prevalence of 0.22–5% [4,5], but exceeds estimates from Germany (96 per 100,000; 0.1%) [10]. Differences in healthcare organization and case ascertainment likely explain this variation.

Whereas OLP typically shows female predominance and CLP a more balanced distribution, this study—examining all subtypes collectively—found near-equal incidence between women (51.4%) and men (48.6%). Aggregation of mucosal and cutaneous forms may mask subtype-specific sex differences. Median age at diagnosis was 58 years, with peak prevalence between 60–69 years, indicating greater burden in older age groups.

Diagnostic Validity

The PPV of LP ICD-10 codes was 78.6%, indicating acceptable diagnostic accuracy with some risk of misclassification. This is slightly lower than a recent study reporting 83.4% for “any LP” and 95.1% when limited to dermatologist-coded diagnoses [13]. That study relied on clinician documentation, whereas our validation required predefined clinical and histopathological criteria assessed independently by two dermatologists, likely producing a more conservative estimate.

Swedish National Patient Register validation studies report PPVs between 72% and 93% [14]. Thus, the observed PPV aligns with accepted registry standards and supports the reliability of LP coding for epidemiological research.

Therapy

Treatment patterns were consistent with European S1 guidelines [15]. Topical corticosteroids were most frequently prescribed, reflecting first-line recommendations. Systemic therapies, phototherapy, and calcineurin inhibitors were less common, consistent with second-line roles. Approximately two-third had no recorded treatment, possibly reflecting mild disease or limited healthcare contact. The relatively high proportion of patients without documented treatment should be interpreted with caution. The registry data only captured prescriptions recorded during the healthcare encounter in which the LP diagnosis was registered and therefore may not reflect treatments prescribed earlier in the disease course or in other clinical settings. Consequently, some patients classified as untreated may have received therapy prior to the recorded visit or may not have required active treatment at that specific time point. Clinical severity and indications were not available, limiting interpretation. Overall, prescribing patterns align with evidence-based practice.

Comorbidity

LP was associated with several autoimmune, metabolic, malignant, psychiatric, and cardiovascular conditions.

Thyroid Disease

Significantly elevated rates of hypothyroidism and autoimmune thyroiditis were observed, consistent with prior studies [10,16]. OLP patients often exhibit anti-TPO and anti-Tg antibodies [17]. A Mendelian randomization study demonstrated a bidirectional relationship between LP and thyroid disease [18], and shared T-cell-mediated immune mechanisms have been proposed [19]. These findings support thyroid dysfunction as a clinically relevant comorbidity warranting consideration in routine assessment.

Hepatitis C

No significant association with HCV was found, likely due to very low case numbers (n = 5). Sweden has low HCV prevalence (0.36% in 2013) [20], limiting statistical power. Prior significant associations were reported in higher-prevalence settings or larger cohorts [10,21,22]. Thus, absence of association here likely reflects low event numbers rather than lack of biological link.

Autoimmune Diseases

Sjögren’s syndrome, systemic lupus erythematosus, and dermatomyositis were significantly associated, consistent with prior findings [23]. However, the association involving dermatomyositis was based on very small case numbers and should therefore be interpreted cautiously despite statistical significance. Rheumatoid arthritis was not significantly associated. Shared T-cell dysregulation [2,24], abnormal type I interferon signaling [23], and overlapping immune-regulatory loci [25] may explain these links. Interestingly, LP showed a significant association with ulcerative colitis but not Crohn’s disease. Although both conditions belong to the spectrum of inflammatory bowel disease, they differ in their immunological profiles and mucosal immune responses [26]. The observed association may reflect shared mechanisms of immune dysregulation, but given the relatively small number of IBD cases in our cohort, this finding should be interpreted cautiously and warrants further investigation.

Metabolic Disorders

Hypertension, dyslipidemia, obesity, and both type 1 and type 2 diabetes were significantly associated with LP. These findings align with previous reports [10,27] and support the concept of systemic metabolic dysregulation in LP. Screening for metabolic comorbidity may therefore be clinically relevant.

Infections

We observed a significant association between LP and condyloma. Previous studies have investigated a possible role of HPV infection in the pathogenesis of lichen planus, particularly mucosal forms such as oral or genital LP. Some meta-analyses have reported an increased prevalence of HPV among patients with oral lichen planus, suggesting that viral infection could potentially act as a trigger or contributing factor in susceptible individuals [28]. However, the evidence remains inconsistent, and the small number of cases in our cohort warrants cautious interpretation.

Increased odds were observed for cancers of the vulva/vagina, penis, lip/oral cavity, and lymphomas, consistent with previous reports [10,29]. However, event numbers were low and confidence intervals wide, warranting cautious interpretation and confirmation in larger prospective cohorts.

Psychiatric and Cardiovascular Disease

LP was associated with depression, consistent with prior research [30], but not anxiety. Increased prevalence of angina pectoris and chronic ischemic heart disease aligns with previous evidence of modest cardiovascular risk [31]. LP may therefore represent a marker of systemic inflammatory and cardiovascular vulnerability.

Strengths and Limitations

Strengths include the large population-based design, comprehensive regional coverage, use of electronic health records, and independent dermatological validation of diagnoses. Adjustment for age and sex strengthened analytical robustness.

Limitations include reliance on ICD-10 coding, unmeasured confounders (e.g., socioeconomic factors, medication exposure), and possible detection bias due to increased healthcare contact among LP patients. A methodological limitation concerns the definition of age in the two study groups. For LP cases, age was defined at the time of first recorded diagnosis, whereas for controls age was defined at the end of the study period. This asymmetry may lead to a age mismatch between groups, as controls may on average be older at characterization than cases at disease onset. Because age is an important confounder for many comorbid conditions, this could potentially inflate associations with age-related diseases. However, all regression analyses were adjusted for age and sex, which likely mitigates this effect. Nevertheless, residual confounding cannot be excluded.

One limitation relates to the large number of comorbid conditions analysed. Logistic regression models were applied to 95 predefined diagnoses, which increases the risk of type I error due to multiple comparisons. Examples include lymphoma, obsessive–compulsive disorder, alcohol-related diagnoses, thyrotoxicosis, thyroiditis, condyloma, systemic lupus erythematosus, and ulcerative colitis. Consequently, results should be interpreted cautiously and considered hypothesis-generating rather than definitive.

Patients with lichen planus may have more frequent healthcare contact, particularly with dermatologists, which could increase the likelihood that comorbid conditions are diagnosed and recorded compared with controls from the general population. This differential ascertainment may lead to overestimation of the observed associations.

An important diagnostic consideration in lichen planus is the distinction from lichenoid reactions, particularly drug-induced lichenoid eruptions, which may clinically and histologically resemble idiopathic LP. Several medications, including antihypertensives, antimalarials, and nonsteroidal anti-inflammatory drugs, have been reported to trigger lichenoid reactions [32]. This issue is particularly relevant in mucosal disease, where differential diagnosis may be challenging. In clinical practice, histopathological examination is often used to support the diagnosis when the presentation is atypical or when mucosal lesions are involved [1]. Because our study relied on registry-based ICD-10 coding and primarily captured cutaneous LP (L43), detailed clinical differentiation between idiopathic LP and lichenoid reactions could not be assessed.

Ethical Considerations

This retrospective registry-based study involved minimal direct risk. Data were pseudonymized, although medical record review required identifiable access. Data handling complied with GDPR and received approval from the Research Data Center in Region Jönköping and the Swedish Ethical Review Authority.

Written informed consent was waived due to the population-level design, retrospective nature, pseudonymization, and minimal risk. Although this limits autonomy, the waiver was considered proportionate and ethically justified.

## 5. Conclusions

This population-based study demonstrates that LP is associated with systemic autoimmune, metabolic, malignant, psychiatric, and cardiovascular conditions, supporting its characterization as a multisystem inflammatory disease. Registry-based case identification showed acceptable validity, and treatment patterns were consistent with current guidelines. These findings highlight the need for holistic clinical management and further prospective studies to clarify mechanisms and optimize care.

## Figures and Tables

**Table 1:** Demographic data for incident patients diagnosed with lichen planus (LP) and the control cohort (year 2013 to 2022).

	Cases			Controls		
	n = 708	f = 364 (51.4%)	m = 344 (48.6%)	n = 360,964	f = 178,384	m = 182,577
Median age (min–max)	58 (9–93)	61 (9–93)	56 (14–93)	41 (0–106)	42 (0–106)	40 (0–103)
Age group	n (%)	n		n (%)	n	
0–<18	12 (1.7)	6	6	78,039 (21.6)	37,963	40,076
18–<30	52 (7.3)	17	35	48,693 (13.5)	22,933	25,760
30–<40	60 (8.5)	26	34	47,663 (13.2)	23,113	24,550
40–<50	109 (15.4)	50	59	42,064 (11.7)	20,618	21,446
50–<60	139 (19.6)	73	66	46,039 (12.8)	22,576	23,463
60–<70	150 (21.2)	83	67	39,340 (10.9)	19,357	19,983
70–<80	130 (18.4)	69	61	36,151 (10.0)	18,413	17,738
80–<90	48 (6.8)	35	13	18,803 (5.2)	10,586	8217
≥90	8 (1.1)	5	3	4169 (1.2)	2825	1344

n = number, f = female, m = male.

**Table 2:** Clinical subtypes of lichen planus among prevalent cases in 2022.

LP Subtype	n (%)
Hypertrophic	66 (7.8)
Bullous	3 (0.4)
Erosive/subacute	57 (6.7)
Ulcerous/erythematous/atrophic	201 (23.6)
Unspecified	657 (77.2)
Total	851 (115.7 *)

* Subtype categories are not mutually exclusive; patients may have received more than one subtype code.

**Table 3:** Physician specialties treating incident LP patients.

Specialty	n (%)
Dermatology	514 (72.6)
Primary care	114 (16.1)
Obstetrics and Gynecology	35 (4.9)
Otorhinolaryngology	28 (4.0)
Urology	5 (0.7)
Internal Medicine	4 (0.6)
Surgery	3 (0.4)
Other	5 (0.7)

**Table 4:** Treatment of prevalent LP patients in 2022.

Therapy	% (n) of Prevalent LP Patients
*Topical therapy*	36.9 (314)
Topical corticosteroids	30.6 (260)
- Weak (group I)	0.6 (5)
- Moderately potent (group II)	3.4 (29)
- Potent (group III)	17.5 (149)
- Very potent (group IV)	8.3 (71)
- Combinations	0.7 (6)
Antifungal	3.5 (30)
Takrolimus/pimecrolimus	2.5 (22)
Topical vitamin D derivates	0.2 (2)
*Intralesional therapy*	0.6 (5)
Triamcinolon	0.6 (5)
*Systemic therapy*	4.3 (37)
Systemic corticosteroids	3.5 (30)
Systemic retinoids	0.8 (7)
Aminoquinolines	(0)
*UV therapy*	1.1 (9)
UVB phototherapy	1.1 (9)
PUVA (photochemotherapy)	(0)
Bucky	(0)
*treatment*	30.3% (258)
*no treatment*	69.7% (593)

**Table 5:** Frequency and odds ratio of comorbidity in prevalent lichen planus (LP) patients compared to control group. Odds ratio is adjusted for age and sex.

Category	Diseases	ICD-10 Code	Lichen Planus 851 (%) 439 Females 412 Males	Controls 360,961 (%) 178,384 Females 182,577 Males	OR (95% CI)	Adjusted OR (95% CI)	Sign.
Malignancies	Malignant neoplasms vulva/vagina	C51, C52	3 (0.7)	78 (0.04)	15.7 (4.9–50.0)	9.4 (2.9–29.9)	***
	Malignant neoplasms penis	C60	3 (0.7)	82 (0.05)	16.3 (5.1–51.9)	10.4 (3.2–33.2)	***
	Malignant melanoma of the skin	C43	11 (1.3)	2493 (0.7)	1.9 (1.0–3.4)	1.2 (0.6–2.1)	ns
	Other malignant neoplasms of the skin	C44	57 (6.7)	12,970 (3.6)	1.9 (1.5–2.5)	1.1 (0.8–1.5)	ns
	Malignant neoplasms of lip, oral cavity, pharynx	C00-C14	10 (1.2)	385 (0.1)	11.1 (5.9–20.9)	7.1 (3.8–13.4)	***
	Hodgkin and Non-Hodgkin lymphoma	C81, C85	4 (0.5)	411 (0.1)	4.1 (1.5–11.1)	2.7 (1.0–7.3)	†
	Leukaemia	C91-C95	5 (0.6)	688 (0.2)	3.1 (1.3–7.5)	2.0 (0.8–4.8)	ns
	Benign neoplasm of other and unspecified intrathoracic organs	D15	1 (0.1)	52 (0.0)	8.2 (1.1–59.1)	5.4 (0.7–39.1)	ns
Metabolic syndrome	Hypertension	I10	409 (48.1)	78,146 (21.6)	3.3 (2.9–3.8)	2.0 (1.7–2.4)	***
	Disorders of lipoprotein metabolism or other lipidemia	E78	294 (34.5)	52,985 (14.7)	3.1 (2.7–3.5)	1.9 (1.6–2.2)	***
	Obesity	E66	161 (18.9)	35,739 (9.9)	2.1 (1.8–2.5)	1.6 (1.3–1.9)	***
	Type 1 diabetes mellitus	E10	30 (3.5)	4950 (1.4)	2.6 (1.8–3.8)	1.9 (1.3–2.8)	***
	Type 2 diabetes mellitus	E11	135 (15.9)	22,831 (6.3)	2.8 (2.3–3.4)	1.8 (1.4–2.1)	***
Psychiatric conditions	Manic episode	F30	1 (0.1)	259 (0.1)	1.6 (0.2–11.7)	1.3 (0.2–9.2)	ns
	Bipolar affective disorder	F31	5 (0.6)	2583 (0.7)	0.8 (0.3–2.0)	0.7 (0.3–1.6)	ns
	Depression	F32, F33	202 (23.7)	54,090 (15.0)	1.8 (1.5–2.1)	1.4 (1.2–1.6)	***
	Persistent mood disorders	F34	10 (1.2)	1491 (0.4)	2.9 (1.5–1.4)	2.4 (1.3–4.5)	**
	Phobic anxiety disorders or other anxiety disorders	F40, F41	194 (22.8)	62,686 (17.4)	1.4 (1.2–1.6)	1.1 (1.0–1.3)	ns
	Obsessive-compulsive disorders	F42	11 (1.3)	2823 (0.8)	1.7 (0.9–3.0)	1.9 (1.0–3.4)	†
	Specific personality disorder	F60	2 (0.2)	1661 (0.5)	0.5 (0.1–2.0)	0.5 (0.1–2.0)	ns
Inflammatory bowel diseases	Crohn’s disease	K50	7 (0.8)	1739 (0.5)	1.7 (0.8–3.6)	1.4 (0.6–2.9)	ns
	Ulcerative colitis	K51	16 (1.9)	2819 (0.8)	2.4 (1.5–4.0)	1.9 (1.1–3.0)	†
Autoimmune diseases	Sjögren’s syndrome	M35.0	13 (1.5)	1154 (0.3)	4.8 (2.8–8.4)	3.0 (1.7–5.2)	***
	Chronic atrophic gastritis	K29.4	2 (0.2)	350 (0.1)	2.4 (0.6–9.8)	1.5 (0.4–6.1)	ns
	Ankolysing spondylitis	M45	1 (0.1)	947 (0.3)	0.4 (0.1–3.2)	0.3 (0.0–2.3)	ns
	Rheumatoid arthritis and other arthritis	M05, M06, M07, M08	12 (1.4)	4304 (1.2)	1.2 (0.7–2.1)	0.8 (0.4–1.4)	ns
	Dermatomyositis	M33	3 (0.4)	73 (0.02)	17.5 (5.5–55.6)	12 (3.8–38.2)	***
	Vasculitis	L95	2 (0.2)	392 (0.1)	2.2 (0.5–8.7)	1.5 (0.4–6.0)	ns
	Systemic Lupus erythematosus	M32, L93	4 (0.5)	411 (0.1)	4.1 (1.5–11.1)	2.8 (1.1–7.6)	†
Lifestyle factors and cardiovascular conditions	Smoking	Z72.0, F17.2	87 (10.2)	16,490 (4.6)	2.4 (1.9–3.0)	1.7 (1.3–2.1)	***
	Alcohol	Z72.1, F10.2	20 (2.4)	3847 (1.1)	2.2 (1.4–3.5)	1.6 (1.0–2.5)	†
	Angina pectoris	I20	59 (6.9)	9979 (2.8)	2.6 (2.0–3.4)	1.6 (1.2–2.1)	***
	Chronic ischemic heart disease	I25	104 (12.2)	15,897 (4.4)	3.0 (2.5–3.7)	2.0 (1.6–2.5)	***
	Arteriosclerosis	I70	19 (2.2)	2257 (0.6)	3.6 (2.3–5.7)	2.3 (1.4–3.6)	***
	Secondary hypertension	I15	1 (0.1)	541 (0.1)	0.8 (0.1–5.6)	0.5 (0.1–3.8)	ns
Infections	Candidiasis of mouth or esophagus	B37, B37.8D	173 (20.3)	25,370 (7.0)	3.4 (2.9–4.0)	2.8 (2.3–3.3)	***
	Candidiasis of skin or other locations	B37.2	20 (2.4)	2481 (0.7)	3.5 (2.2–5.4)	2.6 (1.6–4.0)	***
	Candidiasis of urogenital sites	B37.3, B37.4	63 (7.4)	10,326 (2.9)	2.7 (2.1–3.5)	2.5 (1.9–3.2)	***
	Condyloma	A63	10 (1.2)	2265 (0.6)	1.9 (1.0–3.5)	2.0 (1.1–3.7)	†
Dermatologic conditions	Alopecia areata	L63	14 (1.6)	1589 (0.4)	3.8 (2.2–6.4)	3.6 (2.1–6.0)	***
	Psoriasis vulgaris	L40	90 (10.6)	9079 (2.5)	4.6 (3.7–5.7)	3.3 (2.6–4.1)	***
	Vitiligo	L80	6 (0.7)	1228 (0.3)	2.1 (0.9–4.7)	1.8 (0.8–4.1)	ns
	Pyoderma gangraenosum	L88	1 (0.1)	77 (0.02)	5.5 (0.8–40.0)	3.9 (0.5–28.0)	ns
	Leucoplakia of vagina or vulva	N90.4, N89.4	32 (3.8)	700 (0.2)	20.1 (14.0–28.8)	12.7 (8.7–18.4)	***
	Leucoplakia of vocal cords or larynx	J38.3, J38.7	4 (0.5)	568 (0.2)	3.0 (1.1–8.0)	2.3 (0.9–6.3)	ns
	Lichen sclerosis	L90.0	91 (10.7)	2437 (0.7)	17.6 (14.1–22.0)	11.9 (9.4–14.9)	***
Thyroid disorders	Autoimmune thyroiditis or thyroiditis	E06.0, E06.1, E06.2, E06.3, E06.5	6 (0.7)	857 (0.2)	3.0 (1.3–6.7)	2.4 (1.1–5.3)	†
	Hypothyroidism	E01, E03	82 (9.6)	14,848 (4.1)	2.5 (2.0–3.1)	1.6 (1.3–2.1)	***
	Thyrotoxicosis	E05	21 (2.5)	3760 (1.0)	2.4 (1.6–3.7)	1.6 (1.0–2.5)	†
Liver diseases	Acute and chronic hepatitis B	B16, B18.0, B18.1	3 (0.4)	800 (0.2)	1.6 (0.5–5.0)	1.4 (0.5–4.5)	ns
	Acute and chronic hepatitis C	B17.1, B18.2	5 (0.6)	933 (0.3)	2.3 (0.9–5.5)	1.7 (0.7–4.2)	ns

** = *p*-value < 0.01; *** = *p*-value < 0.001. † Borderline statistical significance (confidence interval close to unity; interpret with caution).

**Table 6:** Diagnosis with no prevalent lichen planus cases in the study population in 2022.

Disease	ICD-10 Code
Autoimmune hepatitis	K75.4
Acute hepatitis B	B16
Primary biliary cirrhosis	K74.3
Multiple sclerosis	G35
Malignant neoplasms in thymus	C37

## Data Availability

The data that support the findings of this study are not publicly available due to legal and ethical restrictions under the General Data Protection Regulation (GDPR) and Swedish data protection laws. The data were obtained from Region Jönköping County and are available from the authors upon reasonable request and with permission from Region Jönköping County and the Swedish Ethical Review Authority.

## Abbreviations

The following abbreviations are used in this manuscript:

LP	Lichen planus
OLP	Oral lichen planus
CLP	Cutaneous lichen planus
PPV	Positive predictive value
ICD-10	International Classification of Diseases, 10th Revision
NPR	National Patient Register
HCV	Hepatitis C virus
GDPR	General Data Protection Regulation
FORSS	Medical Research Council of Southeast Sweden
